# Survival Impact of Preoperative Hemoglobin-to-Red Cell Distribution Width Ratio in Stage I Gastric Cancer Treated with Curative Surgery

**DOI:** 10.31662/jmaj.2025-0599

**Published:** 2026-04-03

**Authors:** Masayuki Urabe, Mami Suzuki, Takahiro Fukai, Yui Hasegawa, Emi Terai, Yoshitaka Kiya, Goki Morizono, Masaya Hiyoshi, Toshiyuki Watanabe, Yojiro Hashiguchi

**Affiliations:** 1 Gastrointestinal Surgery Division, Department of Surgery, Japanese Red Cross Omori Hospital, Tokyo, Japan

**Keywords:** gastric cancer, hemoglobin-to-red cell distribution width ratio, prognosis, surgery

## Abstract

**Introduction:**

The determinants of survival in early-stage gastric cancer (GC), where long-term outcomes are often driven more by non-cancer-related factors than by tumor biology, remain insufficiently defined. We hypothesized that the hemoglobin-to-red cell distribution width ratio (HRR) may serve as a useful prognostic indicator in this setting and evaluated its association with long-term outcomes in stage I GC.

**Methods:**

We retrospectively reviewed the clinicopathological data of 120 consecutive patients with pathological stage I GC who underwent R0 surgical resection. The prognostic value of HRR was assessed using time-dependent receiver operating characteristic analysis and Cox proportional hazards regression. Optimal cutoff values were determined using X-tile software.

**Results:**

Time-dependent receiver operating characteristic analyses demonstrated that HRR consistently yielded higher areas under the curve for overall survival (OS) than the prognostic nutritional index during the first four years after surgery, indicating superior discriminatory performance. In univariate Cox regression analysis, preoperative HRR was significantly associated with OS. In multivariate models adjusted for clinicopathological covariates, low preoperative HRR (≤0.183) remained independently associated with poorer OS (hazard ratio, 8.87; 95% confidence interval, 2.84-27.7; p < 0.001), and this association persisted when HRR was treated as a continuous variable.

**Conclusions:**

Preoperative HRR was a robust prognostic marker for OS in patients undergoing curative surgery for stage I GC. Given its simplicity and accessibility, HRR may represent a practical tool for mortality risk stratification in early-stage GC, a population in which non-cancer-related factors substantially influence survival outcomes.

## Introduction

Gastric cancer (GC) remains a major global health burden and is currently the fifth leading cause of cancer-related mortality, accounting for an estimated 660,000 deaths annually ^(1)^. Although multimodal therapy incorporating perioperative chemotherapy is essential once GC progresses beyond the early stage, recurrence after oncologically adequate resection is infrequent in early-stage disease ^(2)^. In this context, long-term survival is often determined more by comorbid conditions and physiological reserve than by intrinsic tumor aggressiveness ^(3)^. For patients at elevated operative risk, the clinical priority frequently shifts toward optimizing perioperative safety rather than pursuing maximal oncologic radicality. This underscores the need for simple and reliable preoperative tools to predict postoperative survival. Nevertheless, currently available risk-assessment models remain limited in number, methodologically heterogeneous, and often too complex for routine implementation.

Red cell distribution width (RDW), an inexpensive and universally reported component of the complete blood count, quantifies variability in erythrocyte size and reflects multiple biological processes that indicate systemic health. RDW increases in response to impaired iron metabolism, bone marrow dysfunction, and heightened oxidative stress, physiological disturbances closely linked to frailty and poor surgical tolerance ^(4), (5)^. In oncologic settings, RDW is increasingly recognized as a surrogate marker of host inflammatory and nutritional status, both of which critically influence postoperative resilience and long-term survival ^(6)^. The hemoglobin-to-RDW ratio (HRR) further refines this concept by integrating hemoglobin concentration, a proxy for oxygen-carrying capacity and overall metabolic reserve, with anisocytosis ^(7), (8)^. By combining these parameters, HRR reduces the noise inherent in each individual measure and provides a composite index of physiological robustness.

Given this biological and clinical rationale, we hypothesized that preoperative HRR serves as a clinically meaningful prognostic indicator in early-stage GC. To test this hypothesis, we conducted a retrospective cohort analysis to examine the association between preoperative HRR and survival outcomes among patients undergoing curative gastrectomy for stage I GC.

## Materials and Methods

### Study population

Among patients who underwent surgical resection for histologically confirmed GC at our institution between July 2010 and February 2025 without receiving neoadjuvant chemotherapy, 138 cases of pathological stage I GC (pT1N0, pT1N1, and pT2N0) were identified ^(9)^. Eighteen patients were excluded according to the following criteria: cancer of the remnant stomach (n = 3), synchronous malignancies (n = 8), and incomplete laboratory data (n = 7). Cases involving R1/R2 resection, emergency surgery, acute infectious diseases, connective tissue diseases, or a follow-up period shorter than one month were not included. Consequently, 120 patients were eligible for the final retrospective analysis.

Given that this study relied solely on anonymized clinical information collected through routine care, the requirement for written informed consent was waived. The study was conducted in accordance with the principles of the Declaration of Helsinki and the Ethical Guidelines for Medical and Health Research Involving Human Subjects in Japan and was approved by the Institutional Review Board of our institution (Approval No. 24-33).

### Clinicopathological data

The HRR was calculated by dividing the hemoglobin concentration (g/dL) by the RDW standard deviation (fL). In addition to HRR, the preoperative prognostic nutritional index (PNI) was assessed as a representative prognostic marker in early-stage GC ^(10)^. The PNI was calculated using the following formula: serum albumin level (g/L) + 0.005 × total lymphocyte count (10^6^/L). All preoperative laboratory measurements were, in principle, obtained within one week prior to surgery.

Tumor staging was determined according to the eighth edition of the Union for International Cancer Control Tumor, Node, Metastasis classification ^(11)^. Histological subtype was categorized as intestinal or diffuse based on the Lauren classification ^(12)^. Postoperative complications were defined as adverse events occurring within 30 days of surgery and were graded as Clavien-Dindo class III or higher ^(13)^.

All patients were followed for at least five years after surgery or until death, in accordance with the Japanese Gastric Cancer Association guidelines ^(9)^. Postoperative surveillance consisted of routine physical examination, esophagogastroduodenoscopy, computed tomography, abdominal ultrasonography, and standard blood testing performed at intervals consistent with guideline-based protocols. Follow-up evaluations were generally performed every six months during the first three years after surgery and annually thereafter, unless additional assessments were clinically indicated. For individuals who did not attend scheduled follow-up visits, survival and recurrence status were confirmed through structured telephone interviews. Follow-up for the entire cohort was completed in October 2025.

### Statistical analysis

Continuous variables were compared using the Wilcoxon rank-sum test. Overall survival (OS) was defined as the interval from surgery to death from any cause. Time-dependent receiver operating characteristic (ROC) curves for OS prediction based on preoperative PNI and HRR were constructed using the “timeROC” package in R. Optimal cutoff values for PNI and HRR were determined using X-tile software, which selects the threshold that provides the maximal separation of survival outcomes ^(14)^. Survival curves were generated using the Kaplan-Meier method and compared with the log-rank test. The prognostic relevance of each variable was first evaluated using univariate Cox proportional hazards regression analysis. Variables that were statistically significant in univariate analysis, together with clinically important factors judged a priori to be relevant, were subsequently incorporated into multivariate Cox regression models. To ensure model stability and avoid overfitting, the number of covariates included in the multivariate model was restricted based on the number of observed events. Model discrimination for survival stratification was assessed using the Akaike information criterion (AIC) within the Cox proportional hazards framework.

A two-tailed *p-*value < 0.05 was considered statistically significant. All statistical analyses were performed using JMP Student Edition version 18.2.1 (SAS Institute, Cary, NC, USA), X-tile version 3.6.1 (Yale University, New Haven, CT, USA), and R version 4.5.1 (R Foundation for Statistical Computing, Vienna, Austria).

## Results

### Associations between preoperative HRR and clinicopathological features

The relationships between preoperative HRR and clinicopathological variables are shown in Table 1. Preoperative HRR had significant associations with age at surgery (dichotomized at 70 years), American Society of Anesthesiologists physical status, and surgical approach.

**Table 1.** Associations between Preoperative HRR and Clinicopathological Factors.

Variables	#	HRR Median (IQR)	p-Value
Total	120	0.29 (0.23-0.31)	
Age at surgery (years)			
≤ 69	39	0.30 (0.24-0.32)	0.020^*^
≥ 70	81	0.27 (0.22-0.31)	
Gender			
Male	85	0.28 (0.23-0.31)	0.98
Female	35	0.29 (0.22-0.32)	
ASA-PS			
1-2	99	0.29 (0.24-0.32)	0.003^*^
3	21	0.22 (0.20-0.29)	
Main histology			
Intestinal	75	0.28 (0.23-0.32)	0.72
Diffuse	45	0.29 (0.24-0.31)	
Main location			
Upper/middle third	32	0.28 (0.21-0.30)	0.32
Lower third	88	0.29 (0.24-0.32)	
Type of gastrectomy			
Partial	108	0.29 (0.24-0.32)	0.080
Total	12	0.27 (0.19-0.29)	
Surgical approach			
Open	87	0.27 (0.22-0.31)	0.032^*^
Laparoscopic/robotic	33	0.30 (0.27-0.32)	
Preoperative ER			
Absent	98	0.27 (0.22-0.31)	0.17
Present	22	0.30 (0.26-0.31)	
pT classification			
T1	98	0.29 (0.24-0.31)	0.63
T2	22	0.27 (0.22-0.32)	
pN classification			
N0	116	0.29 (0.23-0.31)	0.75
N1	4	0.28 (0.25-0.34)	
Lymphovascular involvement			
Absent	85	0.29 (0.23-0.32)	0.69
Present	35	0.28 (0.24-0.31)	
Postoperative complications			
Absent	106	0.29 (0.23-0.32)	0.92
Present	14	0.29 (0.22-0.31)	

ASA-PS, American Society of Anesthesiologists physical status; ER, endoscopic resection; HRR, hemoglobin-to-red cell distribution width ratio; IQR, interquartile range. ^*^p < 0.05.

Linear regression analysis was carried out to assess the association between preoperative HRR and PNI. The correlation was statistically significant (p < 0.001), and the strength of the association was modest (R^2^ = 0.24). This finding indicated that, from the perspective of potential collinearity, HRR and PNI should be evaluated in separate multivariate models.

### Time-dependent ROC analyses

During the study period, 25 deaths occurred within the cohort, of which only one was attributable to cancer recurrence; the remaining 24 were due to non-cancer-related causes, including pneumonia (n = 8), sudden death (n = 5), other malignancies (n = 3), cardiovascular disease (n = 3), senility (n = 3), and liver cirrhosis (n = 2). The median follow-up duration for surviving patients was 61 months.

**Figure 1.**
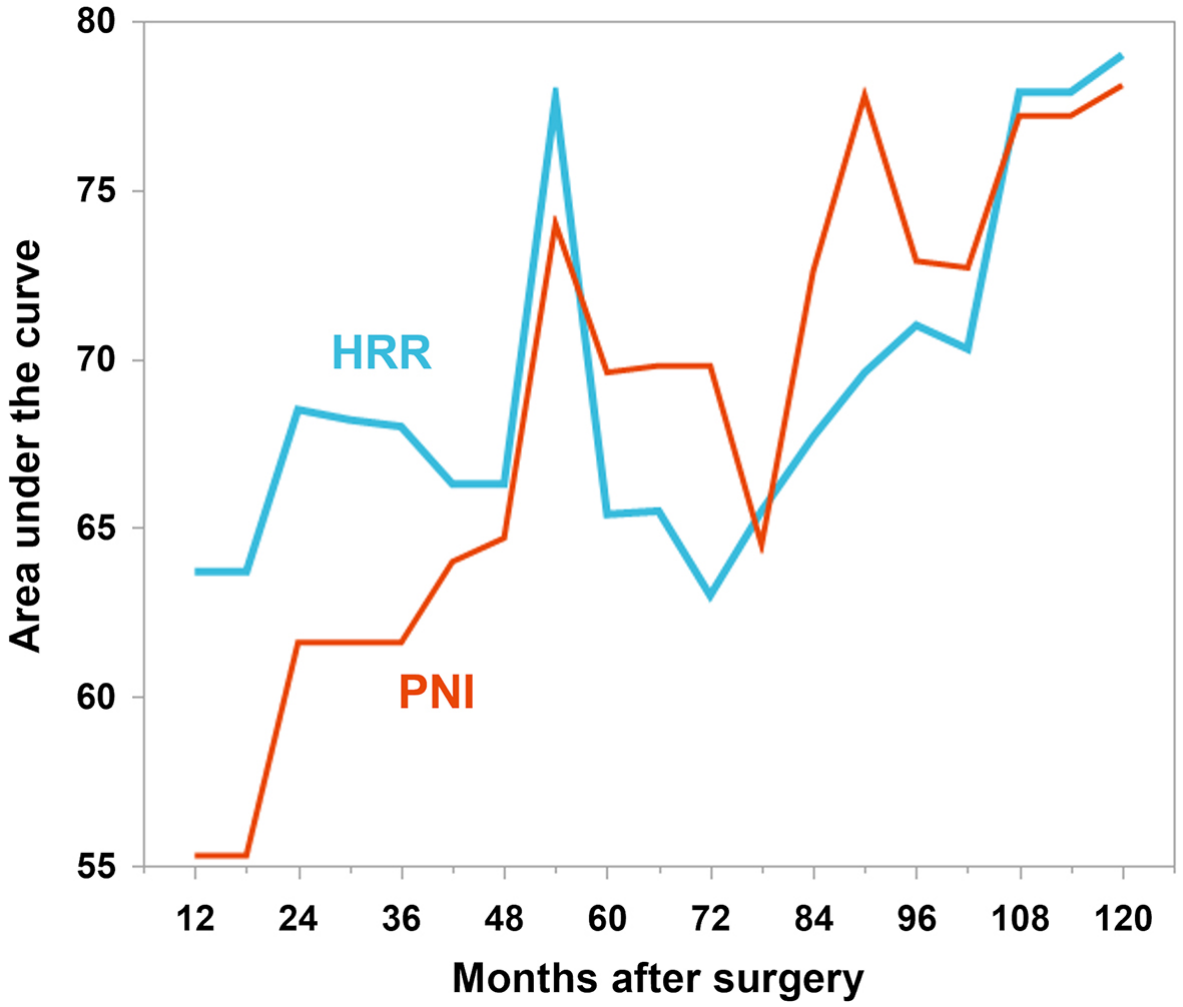
Time-dependent receiver operating characteristic analyses for overall survival.

Time-dependent ROC analyses were conducted, and the area under the curve values for each score were plotted over time for OS (Figure 1). Both preoperative HRR and PNI demonstrated consistent prognostic performance throughout the follow-up period. However, HRR exhibited superior predictive accuracy, particularly during the first four years after surgery, suggesting that HRR may serve as a more reliable preoperative marker for identifying patients at elevated risk of mortality during the early postoperative period.

### Determination of optimal cutoff values using X-tile and Kaplan-Meier survival analyses

**Figure 2.**
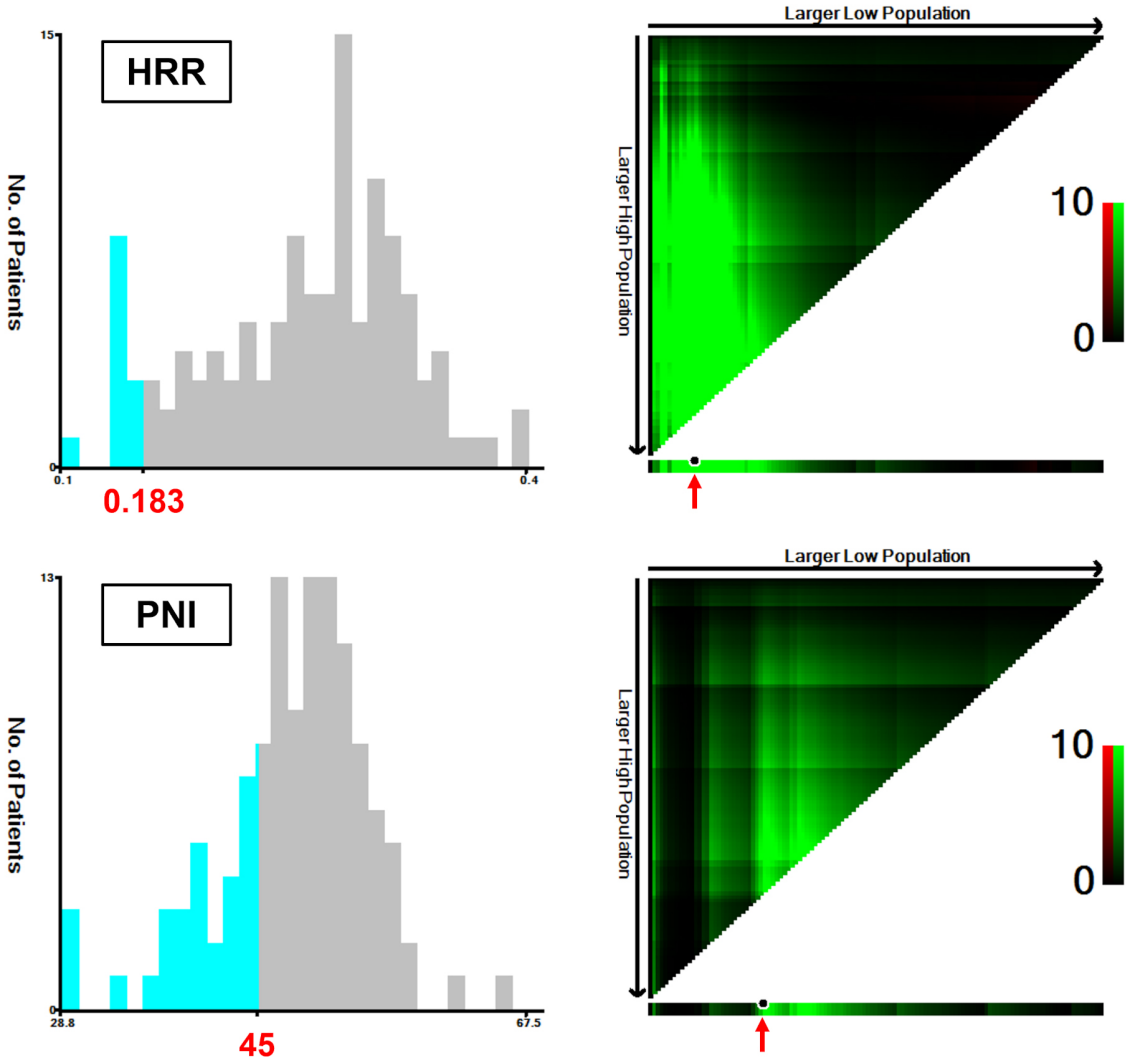
Cut-off value determination for each score employing X-tile software.

**Figure 3.**
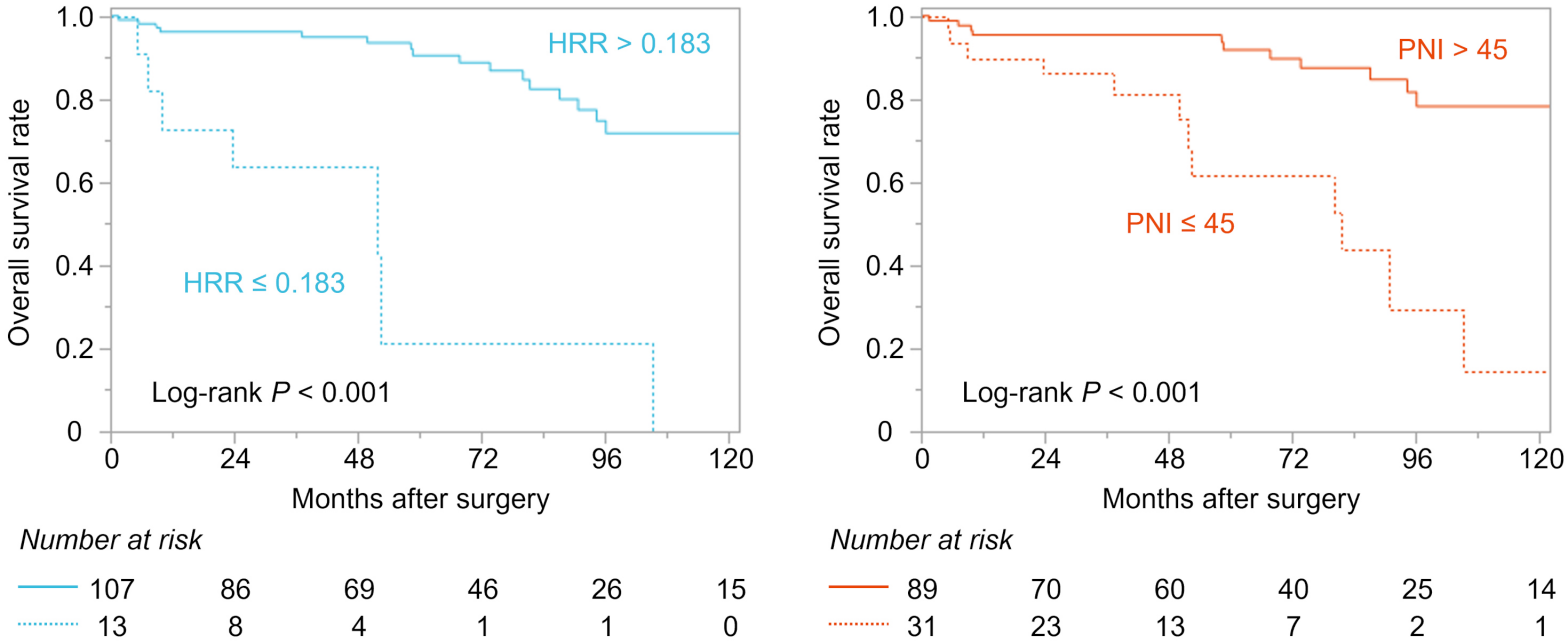
Survival curves for overall survival according to preoperative hemoglobin-to-red cell distribution width ratio and prognostic nutritional index.

Optimal cutoff values for preoperative HRR and PNI were determined using X-tile software based on five-year OS data. The optimal threshold for HRR was 0.183, whereas that for PNI was 45, coincidentally matching the cutoff commonly reported in previous studies (Figure 2) ^(10)^. Patients were subsequently dichotomized according to these thresholds, and Kaplan-Meier survival analyses were performed for OS (Figure 3). Both HRR and PNI demonstrated significant prognostic stratification, with lower values associated with poorer OS (both p < 0.001).

### Cox regression analyses

In univariate Cox regression analyses, both preoperative HRR and PNI were significantly associated with OS, whether analyzed as dichotomized variables or as continuous measures (Table 2). When analyzed as dichotomized variables, the AIC values for OS were 186.1 for HRR and 188.1 for PNI. When treated as continuous variables, the AIC values were 186.8 for HRR and 191.4 for PNI. These results suggest that HRR provided a slightly better univariate model fit for predicting OS compared with PNI.

**Table 2.** Univariate Cox Regression Analyses for Overall Survival.

Variables	Overall survival	
	HR (95% CI)	p-Value
Age at surgery (≥ 70 years)	4.69 (1.38-15.9)	0.013^*^
Gender (male)	2.20 (0.75-6.41)	0.15
ASA-PS (3)	1.22 (0.46-3.28)	0.69
Main histology (intestinal)	1.86 (0.78-4.46)	0.16
Main location (upper/middle third)	2.98 (1.34-6.59)	0.007^*^
Type of gastrectomy (total)	2.92 (1.09-7.81)	0.033^*^
Surgical approach (open)	1.59 (0.54-4.67)	0.40
Preoperative ER (present)	1.01 (0.30-3.42)	0.99
pT classification (T2)	2.11 (0.87-5.13)	0.098
pN classification (N1)	1.17 (0.16-8.77)	0.88
Lymphovascular involvement (present)	1.67 (0.71-3.94)	0.24
Postoperative complications (present)	2.52 (0.94-6.74)	0.065
Preoperative PNI (≤45)	4.84 (2.16-10.8)	< 0.001^*^
Preoperative PNI (per 1-unit decrease)	1.10 (1.04-1.16)	< 0.001^*^
Preoperative HRR (≤0.183)	8.75 (3.47-22.1)	< 0.001^*^
Preoperative HRR (per 0.1-unit decrease)	4.37 (2.04-9.72)	< 0.001^*^

ASA-PS, American Society of Anesthesiologists physical status; CI, confidence interval; ER, endoscopic resection; HR, hazard ratio; HRR, hemoglobin-to-red cell distribution width ratio; PNI, prognostic nutritional index ^*^p < 0.05.

In multivariate Cox regression analyses, the effects of preoperative HRR and PNI on OS were adjusted for age, sex, tumor location, total gastrectomy, pT classification, and postoperative complications. Preoperative lower HRR remained an independent predictor of OS, whether evaluated as a dichotomous variable (hazard ratio, 8.87; 95% confidence interval, 2.84-27.7; p < 0.001) or as a continuous variable (hazard ratio, 4.32 per 0.1-unit decrease; 95% confidence interval, 1.79-11.0; p = 0.001) (Table 3). PNI also retained independent prognostic significance under both analytic approaches. Comparison of AIC values demonstrated that the models incorporating HRR provided a superior fit for predicting OS even after multivariate adjustment.

**Table 3.** Multivariate Cox Regression Analyses for Overall Survival.

Variables	Overall survival		
	HR (95% CI)^*^	p-Value	AIC
Preoperative PNI (≤45)	4.32 (1.71-10.9)	0.002^†^	184.2
Preoperative PNI (per 1-unit decrease)	1.09 (1.01-1.16)	0.013^†^	188.3
Preoperative HRR (≤0.183)	8.87 (2.84-27.7)	<0.001^†^	181.5
Preoperative HRR (per 0.1-unit decrease)	4.32 (1.79-11.0)	0.001^†^	183.1

AIC, Akaike information criterion; CI, confidence interval; HR, hazard ratio; HRR, hemoglobin-to-red cell distribution width ratio; PNI, prognostic nutritional index ^*^Adjusted with age (≥70 years), gender, tumor location (upper/middle third), total gastrectomy, muscular invasion, and postoperative complications. ^†^p < 0.05.

## Discussion

In the present study, we demonstrated that preoperative HRR independently predicted long-term outcomes in patients with stage I GC, a population in which mortality is more often affected by non-cancer-related causes than by tumor progression ^(2), (3)^. In our cohort, non-GC-related deaths accounted for 24 of the 25 events, indicating that competing risks in the survival analysis were minimal. Although the findings of this study do not provide a definitive explanation for the biological behavior of GC, they are particularly relevant in the context of the increasingly aging GC population, in which non-oncologic comorbidities substantially contribute to overall survival. In Japan, somewhat unexpectedly, individuals aged 80 years and older now account for the greatest share of GC-related deaths, representing roughly half of all GC deaths nationwide ^(15)^.

Previous studies have demonstrated associations between HRR and prognosis in malignancies, including esophageal cancer, pancreatic cancer, and hepatocellular carcinoma ^(16), (17), (18)^. Notably, a prognostic relationship has also been reported in patients with GC undergoing neoadjuvant chemotherapy ^(19)^; however, the analyses were restricted to advanced-stage disease. To our knowledge, the present study is the first to specifically examine HRR in the context of early-stage GC, thereby underscoring the novelty and potential clinical relevance of our findings.

The biological rationale for HRR as a prognostic marker lies in its integration of two complementary hematologic parameters. Hemoglobin reflects systemic oxygen-carrying capacity and overall physiological reserve, whereas RDW serves as a sensitive indicator of chronic inflammation, nutritional deficiency, oxidative stress, and age-related hematopoietic dysregulation ^(7), (8)^. Low HRR thus identifies individuals in whom impaired oxygen delivery coexists with heightened inflammatory or nutritional stress, capturing a state of diminished global biological resilience. Such vulnerability has been consistently linked to mortality, particularly from non-cancer-related causes. In early-stage GC, where tumor-specific mortality is low following curative resection, host factors such as frailty, chronic disease burden, and systemic physiological reserve are likely to play a predominant role in determining long-term survival ^(3)^. Accordingly, HRR may serve as an accessible, integrated surrogate for non-oncologic susceptibility, explaining its robust association with OS in our cohort.

Other factors reflecting systemic vulnerability, such as the PNI, have similarly been reported as prognostic in early-stage GC ^(10)^. However, HRR offers distinct practical advantages. Unlike PNI, which requires biochemical parameters such as albumin and lymphocyte counts, HRR can be readily calculated using routine complete blood count data alone, making it widely available and cost-effective. In our cohort, HRR demonstrated superior predictive performance compared with PNI, as shown by time-dependent ROC analyses over the first four years postoperatively and by lower AIC values in Cox regression models. Biologically, this incremental advantage may reflect HRR’s capacity to capture both systemic reserve (hemoglobin) and hematopoietic or immune stress (RDW), whereas PNI primarily assesses nutritional and immunologic status. Consequently, HRR provides a more comprehensive index of vulnerability in early-stage GC.

From a clinical perspective, HRR may inform personalized treatment strategies for patients identified as high risk. In such individuals, surgical planning might prioritize safety and minimal invasiveness over oncologic radicality. For example, in patients undergoing endoscopic submucosal dissection with non-curative extent but low predicted nodal risk, additional resection might be safely deferred ^(20)^. Similarly, in patients undergoing upfront gastrectomy, reducing the extent of lymphadenectomy or tailoring the resection range may decrease surgical invasiveness, for example, through laparoscopic and endoscopic cooperative surgery for local tumor excision and/or sentinel lymph node sampling ^(21), (22)^. Thus, HRR may provide a practical framework for tailoring treatment intensity according to overall physiological resilience, particularly in elderly patients or those with significant comorbidities.

This study has several limitations. Its retrospective design and single-institution setting introduce the potential for selection bias. The study period spanned more than a decade, during which surgical techniques and perioperative management strategies may have evolved, potentially influencing outcomes despite multivariate adjustments. Furthermore, given that nearly all observed deaths were attributable to non-cancer-related causes, the clinical relevance of this marker in GC warrants careful interpretation. Finally, although HRR appears to reflect non-oncologic vulnerability, this association remains speculative at present, and its applicability to treatment strategies cannot be definitively determined. The cutoff value was derived using X-tile optimization within a single retrospective cohort, raising concerns regarding potential overfitting. To ensure the generalizability of our findings, prospective external validation and mechanistic studies are warranted to further elucidate the pathways linking hematologic parameters with long-term survival in early-stage GC.

In summary, we have demonstrated that preoperative HRR robustly predicts OS in patients with pathological stage I GC. HRR may serve as a valuable tool to identify patients with early-stage GC who are at elevated risk of mortality, enabling the implementation of targeted management strategies and supporting the provision of optimally tailored therapeutic interventions.

## Article Information

### Author Contributions

Masayuki Urabe contributed to the conception and design of the study. All authors acquired data. Masayuki Urabe performed data interpretation and drafted the manuscript. Yoshitaka Kiya, Goki Morizono, Masaya Hiyoshi, Toshiyuki Watanabe, and Yojiro Hashiguchi critically revised the manuscript. All authors read and approved the final version prior to submission.

### Conflicts of Interest

None

### Approval by the Institutional Review Board

This study was approved by the institutional ethics committee of the Japanese Red Cross Omori Hospital (Identification 24-33).

### Informed Consent

Written informed consent was waived because of the retrospective design.
